# Hepatoprotective Effects of the *Cichorium intybus* Root Extract against Alcohol-Induced Liver Injury in Experimental Rats

**DOI:** 10.1155/2021/6643345

**Published:** 2021-06-16

**Authors:** Jihee Kim, Min-Jeong Kim, Jin-Ho Lee, Keunjung Woo, Minah Kim, Tack-Joong Kim

**Affiliations:** ^1^Division of Biological Science and Technology, College of Science and Technology Convergence, Yonsei University, Wonju 26493, Republic of Korea; ^2^Research & Development Center, Doctor TJ Co. Ltd., Wonju 26493, Republic of Korea

## Abstract

The effects of the *Cichorium intybus* root extract (Cii) on alcohol-induced liver disease were investigated using Chang liver cells and male Sprague Dawley rats. Silymarin, a liver-protective agent, was used as a positive control. In cell experiments, after 24 h of treatment with the extract, no cytotoxicity was noted, and death by alcohol was avoided. Migration of Chang liver cells increased after exposure to the extract at a concentration of 400 *μ*g/mL. In animal experiments, alcohol was injected into 6-week-old rats for 1, 3, and 50 days. Oral administration of the drug was performed 30 min before alcohol administration. The control was treated with distilled water, and the drug groups were administered EtOH (40% EtOH + 2.5 mL/kg), EtOH + Cii L (low concentration, 2 mg/kg), EtOH + Cii H (high concentration, 10 mg/kg), or EtOH + silymarin (100 mg/kg). Increased liver weight was observed in the alcohol group, as were increased blood-alcohol concentration and liver damage indicators (glutamic oxalacetic transaminase (GOT), glutamic pyruvate transaminase (GPT), and triglycerides (TG)), decreased alcoholysis enzymes (ADH and ALDH), and increased CYP2E1. In the Cii treatment group, liver weight, blood-alcohol concentration, liver damage indicators (GOT, GPT, and TG), and CYP2E1 were decreased, while alcoholysis enzymes (ADH and ALDH) were increased. The degree of histopathological liver damage was compared visually and by staining with hematoxylin and eosin and oil red O. These results indicated that ingestion of Cii inhibited alcohol-induced liver damage, indicating Cii as a useful treatment for alcohol-induced liver injury.

## 1. Introduction

Alcohol consumption imposes a considerable burden on human health, society, and economies. According to the World Health Organization, 3 million deaths worldwide are caused each year by misuse of alcohol [1]. Excessive consumption of alcohol can lead to acute poisoning and damage to the gastrointestinal tract, pancreas, liver, and cardiovascular system, which can cause other diseases, injuries, and metabolic disorders [2]. The liver, which has a high regenerative capacity and complex functions [3], is called the “silent organ” because 70–80% of alcohol-induced damage does not produce symptoms in the early stages. Therefore, liver disease is often detected only after it becomes a serious condition [4]. The liver is the main organ of alcohol metabolism and the target organ of alcohol injury. Various factors have been identified in the mechanism through which alcohol causes liver disease.

Alcoholic liver disease (ALD) is the leading cause of death from alcohol. It encompasses a broad spectrum of morphological conditions ranging from minimal injury to advanced liver damage [5] and includes categories such as alcoholic fatty liver, hepatitis, and cirrhosis. Alcohol can be toxic when ingested, and the alcohol metabolite acetaldehyde is highly toxic in vivo, affecting multiple organs and causing physiological effects to produce various metabolic diseases [6].

Alcohol metabolism involves both alcohol and acetaldehyde [7]. Alcohol is metabolized through the alcohol dehydrogenase (ADH) pathway in cytosol, the catalase pathway in peroxisomes, and the microsomal ethanol-oxidizing system pathway in the microsome to produce acetaldehyde [8]. These three paths in different subcellular organelles perform complementary alcohol metabolism. Converted acetaldehyde is excreted as acetic acid and CO₂ by aldehyde dehydrogenase (ALDH). Acetate is converted into acetyl coenzyme A and used to generate energy through the tricarboxylic acid (TCA) cycle or to synthesize cholesterol and fatty acids [9]. Alcohol metabolism is increased in chronic alcohol intake, resulting in increased oxygen consumption and active oxygen, which decreases antioxidants and creates partial hypoxia and necrosis of the liver [10, 11]. The plasma malondialdehyde reaction, which is promoted by radicals produced during alcohol metabolism, can greatly increase lipid and cholesterol contents in the liver to destroy liver cells.

Despite advances in the understanding of ALD, little progress has been made in terms of treatment [12]. Liver transplantation is often not an effective option because of the shortage of donors and the technical challenges of the surgery. It is necessary to identify and develop approaches that can slow the rate of ALD outbreaks. Oxidative stress and lipid metabolites are considered the main mechanisms in ALD pathogenesis [13], suggesting antioxidants and drugs that inhibit lipid metabolism as potential treatments for liver damage induced by alcohol. *Cichorium intybus*, a dicotyledon belonging to the Compositae family of perennial plants, is effective in liver improvement and vision recovery, and increasing chicory intake has been reported to significantly increase the number of beneficial bacteria among intestinal bacteria [14]. *Cichorium intybus* root extract (Cii) treatment also improves blood circulation by facilitating flow through veins and arteries and exchange between fluids. The Cii is used extensively, either dried or immediately after harvest. In a Korean patent, the herb is recognized as a preventative for influenza and as a treatment for muscle damage. Multiple investigations have been carried out on the physiological activity of Cii, but its protective effects against alcoholic liver injury and the underlying mechanisms remain unknown.

The objective of the present study was to explore the protective effects of Cii in alcoholic liver injury and the possible underlying mechanisms. We also attempted to confirm that Cii is safe for human consumption, offers effective protection from liver damage induced by alcohol and relief from hangovers, and can be a useful treatment for liver toxicity caused by chronic alcohol consumption.

## 2. Materials and Methods

### 2.1. *Cichorium intybus* Root Extract (Cii)

*Cichorium intybus* root was cultivated according to the Good Agricultural Practice (GAP) of the Rural Development Administration (RDA) and harvested in Eumseong, North Chungcheong Province (GPS: E 128° 62′ N36° 56′). After 48 h of reaction and filtration at 150 rpm, 99% ethanol was added to 50 g of *Cichorium intybus* root samples and 50 g of stem samples. A powder of frozen and dried extract was concentrated and used in analysis.

### 2.2. Cell Culture

Chang liver cells were obtained from the American Type Culture Collection (ATCC). All cell lines were grown in modified Eagle's medium (MEM, Welgene, Korea) supplemented with 10% fetal bovine serum (Access Biologicals, USA) and 1% penicillin-streptomycin (Sigma, USA). Chang liver cells were incubated at 37°C in a humidified atmosphere of 5% CO_2_. Trypsin-EDTA solution (Sigma, USA) was used to detach cells. Cells were washed with phosphate-buffered saline (PBS, Gibco, USA).

### 2.3. Cell Proliferation Assay

Chang liver cell proliferation was determined by the EZ-Cytox assay. For the proliferation assay, Chang liver cells (1 × 10^5^ cells/mL) were seeded in MEM in a 96-well cell culture plate at 37°C for 24 h. After supernatant removal, Chang liver cells were washed twice using PBS. The medium was replaced with serum-free MEM containing Cii (0, 50, 100, 200, and 400 *μ*g/mL). Chang liver cells were incubated for 24 h, and 15% EtOH (Merck, Germany) was added to each well, after which the liver cells were incubated for 1 h 40 min at 37°C. A solution of 10% EZ-Cytox (Biomax, Korea) was added to each well, and Chang liver cells were incubated for 1 h at 37°C. Absorbance was assessed at 450 nm with an ELx800 microplate reader (BioTek, USA).

### 2.4. Cell Migration Assay

For the migration assay, Chang liver cells (1 × 10^5^ cells/mL) were seeded in MEM in a 6-well cell culture plate at 37°C for 24 h. The cell monolayer was manually scratched with a yellow plastic pipette tip and treated with Cii (400 *μ*g/mL) or MEM (control). An inverted microscope equipped with a digital camera was used to obtain images of wound healing at different timepoints for each treatment under magnification. Wound closure was monitored at 48 h, until the borders of the wound could no longer be identified.

### 2.5. DPPH Assay

The antioxidant effect of Cii was determined with a 2, 2-diphenyl-1-picryl-hydrazyl-hydrate (DPPH, Sigma, USA) assay at a concentration of 0.2 mM in an alcohol solution and reacted for 30 min at room temperature. Absorbance was assessed at 517 nm with the ELx800 microplate reader (BioTek, USA).

### 2.6. Animal Experiment

**Figure 1:**
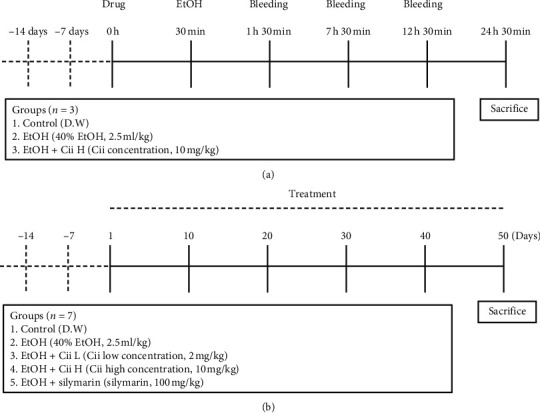
Schematic diagram of the experimental protocol in SD rats. (a) After 2 weeks of acclimation, the rats received alcohol (40%, 2.5 mL/kg) and Cii by oral injection for 1 and 3 days. Cii was administered at 30 min before EtOH injection. The image shows results at 1, 7, 12, and 24 h after the EtOH challenge. (b) After 2 weeks of acclimation, the rats received alcohol (40%, 2.5 mL/kg) by oral injection for 50 days.

Male Sprague Dawley rats (4 weeks old) were purchased from Orient Bio (Korea). The rats were housed in the animal quarters at Yonsei University. The experimental procedures were approved by the Institutional Animal Care and Use Committee (IACUC, YWCI-201908-010-02), which certifies Yonsei University (Wonju, Korea). Cells were acclimated for 14 days at 20–22°C and a relative humidity of 40–50% with a 12 h light/dark cycle. In the short-term administration portion of the experiment, rats were randomly divided into 3 groups (*n* = 3): control, EtOH, and EtOH + Cii (10 mg/kg). After acclimation, the rats received alcohol (40%, 2.5 mL/kg) (Merck, Germany) and Cii by oral injection for 1 and 3 days (Figure 1(a)). For the long-term administration experiment, the rats were randomly divided into 5 groups (*n* = 7): control, EtOH, EtOH + Cii (2 mg/kg), EtOH + Cii (10 mg/kg), and EtOH + silymarin (100 mg/kg). After acclimation, the rats received alcohol (40%, 2.5 mL/kg) (Merck, Germany) by oral injection for 50 days (Figure 1(b)). Each drug was administered orally 30 min before each administration of alcohol. The control group received only distilled water. On the 50th day, the rat was sacrificed at 6 h after the final ethanol challenge, blood was withdrawn, and serum was separated from blood by centrifuging at 3000 rpm for 10 min. The serum was used to estimate blood-alcohol concentration and levels of glutamic oxalacetic transaminase (GOT), glutamic pyruvate transaminase (GPT), and triglycerides (TG). The liver was dissected after perfusion; one part was used for histopathological study and another for additional evaluation. The serum and liver tissue were immediately stored at −70°C.

### 2.7. Determination of Blood-Alcohol Concentration in Rats

Blood was collected, and serum was separated by centrifugation (4°C, 3000 rpm, 10 min). The blood-alcohol concentration was determined according to instructions provided in the reagent kits, which called for use of an NAD-ADH Reagent Multiple Test Vial (Sigma-Aldrich, USA) and 16.0 mL of 0.5 M glycine buffer (pH 9). Next, 3.0 mL of the prepared NAD-ADH reagent was added to labeled test tubes along with 1 *μ*L of serum. The solution was incubated for 10 min at temperatures between 22°C and 37°C and then transferred to cuvettes for absorbance measurement at 340 nm.

### 2.8. Determination of the Rat Liver Index

Six hours after the final ethanol challenge, the body weight of each rat was measured. The liver was removed, and its wet weight was weighed. The liver index value was calculated using the following formula: liver index (%) = (liver weight)/(body weight) × 100.

### 2.9. Determination of Serum GOT, GPT, and TG Levels in Rats

Blood was collected, and serum was separated by centrifugation (4°C, 3000 rpm, 10 min). The levels of serum GOT, GPT, and TG were determined according to instructions provided in reagent kits. An Asan set GOT assay kit (Asan Pharm, Korea) was used to analyze serum GOT, while an Asan set GPT assay kit (Asan Pharm, Korea) was used to analyze serum GPT. A Triglyceride Quantification Kit (Sigma-Aldrich, USA) was used to analyze serum TG. Between 2 and 50 *μ*L of the sample was added to duplicate 96-well plates. The samples were brought to a final volume of 50 *μ*L with a triglyceride assay buffer. A 50 *μ*L master mix was required for each reaction, and the plate was incubated for 60 min at room temperature in the dark. For colorimetric assays, absorbance was measured at 570 nm.

### 2.10. Determination of Liver Tissue ADH, ALDH, and TG Levels in Rats

Liver tissues were stored immediately at −70°C. The levels of liver ADH, ALDH, and TG were determined according to instructions provided in the reagent kits. An Alcohol Dehydrogenase Activity Assay Kit (Sigma-Aldrich, USA) was used to analyze liver ADH. An Aldehyde Dehydrogenase Activity Colorimetric Assay Kit (Sigma-Aldrich, USA) was used to analyze liver ALDH. A 50 mg sample of tissue was homogenized in 200 *μ*g of ice-cold ADH or ALDH assay buffer and was centrifuged at 13,000 g for 10 min to remove insoluble material. Samples were brought to a final volume of 50 *μ*L with ADH or ALDH assay buffer. Next, 100 *μ*L of the appropriate reaction mix was required for each reaction. The plates were incubated at 37°C, and absorbance measurements were performed at 450 nm after 5 min. A Triglyceride Quantification Kit (Sigma-Aldrich, USA) was used to analyze liver TG. Samples of tissue were homogenized in a 1 mL solution of 5% Nonidet P 40 Substitute (Sigma, USA), heated slowly to 80–100°C in a water bath for 5 min or until Nonidet P 40 became cloudy, and then allowed to cool to room temperature. This process was repeated once to solubilize all triglycerides. The samples were centrifuged for 2 min at top speed to remove insoluble material and diluted 10-fold with water before the assay. Next, between 2 and 50 *μ*L samples were added to duplicate wells of a 96-well plate. The samples were brought to a final volume of 50 *μ*L with a TG assay buffer. A master mix of 50 *μ*L was required for each reaction. Plates were incubated for 60 min at room temperature in the dark. For colorimetric assays, absorbance was measured at 570 nm.

### 2.11. Western Blotting

For western blot analysis, polypeptides in whole-tissue lysates were resolved by sodium dodecyl sulfate polyacrylamide gel electrophoresis and transferred to the polyvinyl difluoride (PVDF) membrane. The PVDF membrane was blocked for 1 h in Tris-buffered saline containing 0.1% Tween 20 (TBS-T) with 5% skim milk powder. The blocked membrane was incubated overnight in 4°C with each primary antibody (1 : 5000). The primary antibodies used were anti-cytochrome P450 2E1 (Abcam, UK) and anti-*β*-actin (Cell Signaling, USA). After incubation with the first antibody, the membranes were washed thrice with TBS-T and incubated for 2 h with each secondary antibody at room temperature (1 : 5000). Each protein signal was detected using enhanced chemiluminescence (ECL) reagent, and each image was analyzed with ImageQuant LAS 4000 (GE Healthcare, Buckinghamshire, UK).

### 2.12. Histopathological Observation of Liver Tissues

The liver tissues of rats were fixed in 10% formalin (Biosesang, Korea) and stabilized in 10, 15, or 20% sucrose (Sigma, USA) solutions. To embed fixed livers in a cryomold, an optimal cutting temperature compound (FSC 22 Clear, Leica Biosystems, Wetzlar, Germany) was used. The cryotissues were sectioned into slices (20 *μ*m thick) and stained with hematoxylin (BBC Biochemical, USA) and eosin (Biochemical, USA) (H&E). Histopathological changes in liver tissues were visualized under an Olympus DP80 microscope. Sections were also cut into slices (17 *μ*m thick) and stained with oil red O (Sigma, USA). Histopathological changes of liver tissues were visualized with an Olympus DP80 microscope.

### 2.13. Statistical Analysis

Experimental results were expressed as the mean ± standard error of the mean. Statistical analysis of all the data was performed by one-way analysis of variance (one-way ANOVA) and multiple comparisons followed by a Tukey–Kramer post hoc analysis; *P* < 0.005 and *P* < 0.001 were considered significant and highly significant, respectively.

## 3. Results

### 3.1. Effects of Cii on Chang Liver Cell Proliferation

**Figure 2:**
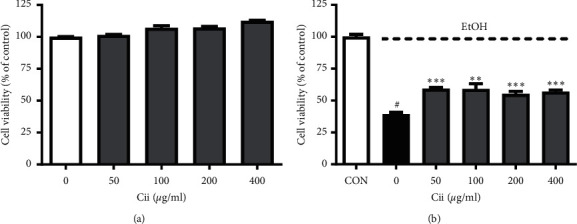
Cytotoxicity of Cii in EtOH-treated Chang liver cells. (a) Chang liver cells were pretreated with various concentrations (50, 100, 200, and 400 *μ*g/mL) of Cii after 24 h of incubation. Cell viability was assessed using the EZ-Cytox assay at 450 nm (*n* = 6). (b) Effect of Cii on cell viability in EtOH-treated Chang liver cells. Chang liver cells were pretreated with various concentrations (50, 100, 200, and 400 *μ*g/mL) of Cii after 24 h of incubation, followed by 15% EtOH for 1 h. Cell viability was assessed using the EZ-Cytox assay at 450 nm. Data were expressed as mean ± S.E.M. (*n* = 6). ^#^*P* < 0.001, CON vs. Cii (0 *μ*g/mL); ^*∗∗*^*P* < 0.005, Cii (0 *μ*g/mL) vs. Cii (100 *μ*g/mL); ^*∗∗∗*^*P* < 0.001, Cii (0 *μ*g/mL) vs. Cii (50, 200, and 400 *μ*g/mL).

An EZ-Cytox assay was used to determine the cytotoxicity of Cii in Chang liver cells. Our sample did not show any cytotoxic effects on cell viability at 50–400 *μ*g/mL (Figure 2(a)). To assess the liver cell regeneration rate of the Cii, it was applied to a Chang liver cell, and cell death was induced using 15% alcohol. As shown in Figure 2(b), treatment of Chang liver cells with 15% alcohol potently decreased cell viability from 100.00 ± 1.88 to 39.11 ± 1.71%. Cii significantly regenerated the alcohol-induced cell viability with 59.11 ± 1.20, 58.89 ± 4.32, 55.04 ± 2.10, and 56.80 ± 1.38% by 50, 100, 200, and 400 *μ*g/mL, respectively.

### 3.2. Effects of Cii on Chang Liver Cell Migration and Invasion

**Figure 3:**
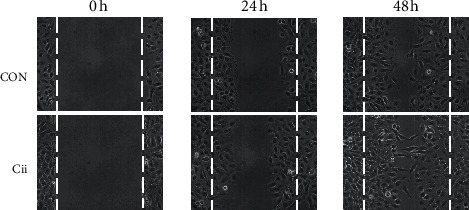
Effect of Cii on Chang liver cell migration. Scratch wound healing assays were performed on Chang liver cells treated with Cii at a concentration of 400 *μ*g/mL to determine the cell migration ability. Scratch wounds in Chang liver cells were shown at time 0 h and represented wound status at 24 h after initiation of the scratch, when the cells were treated with the vehicle control or Cii. Wounds were evaluated at 24 and 48 h after Cii administration.

Cell migration occurs during development or healing of a wound. We conducted an experiment to verify visually whether Cii can protect cells against alcohol-induced damage. Greater cell migration was confirmed in the Cii treatment group compared with the control group, indicating protective effects against damage such as scratches (Figure 3).

### 3.3. Antioxidant Effects of Cii

**Figure 4:**
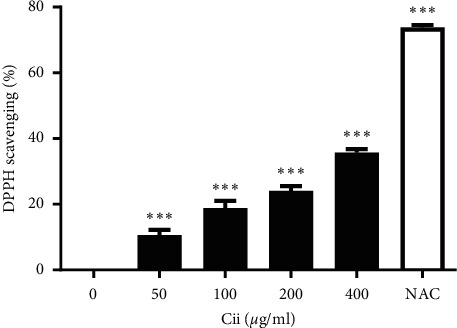
Antioxidant effect of Cii. The antioxidant effect of Cii was determined with a DPPH assay at a concentration of 0.2 mM in an alcohol solution. Absorbance was assessed at 517 nm with the ELx800 microplate reader. Data were expressed as mean ± S.E.M. (*n* = 6). ^*∗∗∗*^*P* < 0.001.

The antioxidant effects of Cii were determined by measuring the degree of DPPH radical erasing, which revealed an increase, suggesting a concentration-dependent antioxidant ability of Cii (Figure 4).

### 3.4. Effects of Cii on Blood-Alcohol Concentration in Rats with Alcohol-Induced Liver Injury

**Figure 5:**
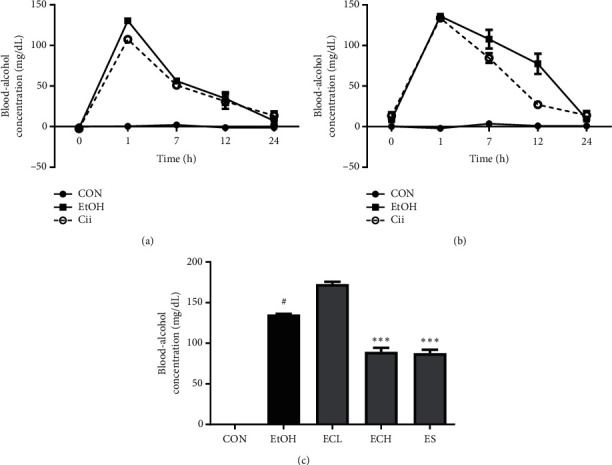
Effect of Cii on blood-alcohol concentration in rats. (a, b) After 2 weeks of acclimation, the rats received alcohol (40%, 2.5 mL/kg) and Cii by oral injection for (a) 1 and (b) 3 days. The rats were randomly divided into 3 groups (*n* = 3): CON (distilled water), EtOH (40% EtOH, 2.5 mL/kg), and Cii (EtOH + Cii concentration, 10 mg/kg). Cii was administered at 30 min before EtOH injection. At 1, 7, 12, and 24 h after the EtOH challenge, blood was collected. (c) After 2 weeks of acclimation, the rats received alcohol (40%, 2.5 mL/kg) by oral injection for 50 days. The rats were randomly divided into 5 groups (*n* = 7): CON (distilled water), EtOH (40% EtOH, 2.5 mL/kg), ECL (EtOH + Cii low concentration, 2 mg/kg), ECH (EtOH + Cii high concentration, 10 mg/kg), and ES (EtOH + silymarin, 100 mg/kg). Data were expressed as mean ± S.E.M. (*n* = 7). ^#^*P* < 0.001, CON vs. EtOH; ^*∗∗∗*^*P* < 0.001, EtOH vs. ECH or ES.

In the short-term administration experiment, the blood-alcohol level tended to decrease faster in the Cii groups than in the alcohol group. In a 1-day animal experiment, the blood-alcohol concentration was 130.44 ± 3.55 and 107.48 ± 2.83 mg/dL at 1 h in EtOH and Cii groups, respectively (Figure 5(a)). In a 3-day animal experiment, the respective blood-alcohol concentration was 107.82 ± 11.59 and 84.47 ± 5.98 mg/dL at 7 h and 77.48 ± 12.63 and 27.11 ± 3.64 mg/dL at 12 h (Figure 5(b)). These results suggest that Cii promotes fast decomposition of alcohol. In the long-term administration experiment, the blood-alcohol concentration increased significantly in the alcohol group compared with the normal group and was significantly reduced in the ECH group compared with the alcohol group. The blood-alcohol concentration was 135.40 ± 0.87, 181.00 ± 6.06, 89.48 ± 4.96, and 87.71 ± 4.36 mg/dL in the EtOH, ECL, ECH, and ES groups, respectively (Figure 5(c)). These results suggest that Cii promotes alcohol decomposition.

### 3.5. Effect of Cii on the Liver Index in Rats with Alcohol-Induced Liver Injury

**Figure 6:**
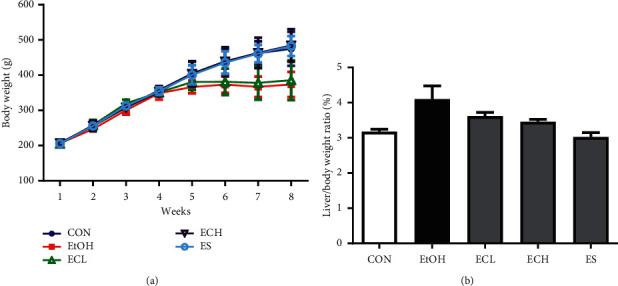
Body weight and ratio of liver weight. CON: distilled water, EtOH: 40% EtOH, 2.5 mL/kg, ECL: EtOH + Cii low concentration, 2 mg/kg, ECH: EtOH + Cii high concentration, 10 mg/kg, and ES: EtOH + silymarin, 100 mg/kg. Data were expressed as mean ± S.E.M. (*n* = 7). (a) Body weight. (b) Liver/body weight ratio.

The effect of Cii on alcohol-induced liver index values of rats was higher in the EtOH group compared with the normal group. Treatment with Cii at 2 and 10 mg/kg reduced the alcohol-induced increase in the liver index compared with the EtOH group. The liver index in the Cii and silymarin group decreased, but no significant difference was evident compared with the EtOH group. The results showed that Cii can reduce the increase in the liver index dose dependently in mice with alcohol-induced liver injury (Figure 6).

### 3.6. Effects of Cii on Serum GOT, GPT, and TG Levels in Rats with Alcohol-Induced Liver Injury

**Figure 7:**
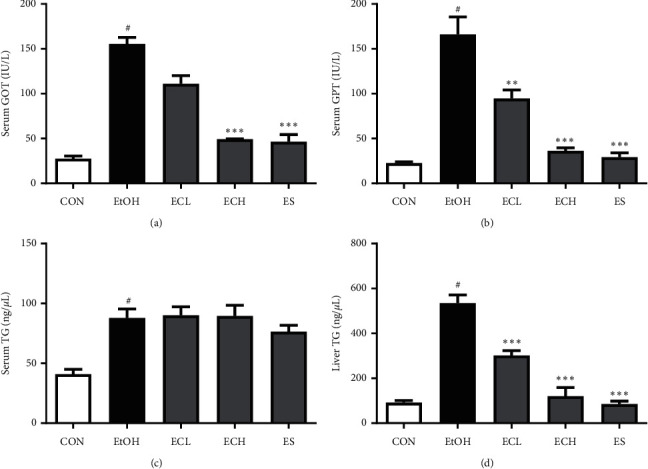
Effects of Cii on GOT, GPT, and TG levels in alcohol-induced rats. The levels of serum GOT, GPT, and TG and liver TG were determined by commercial reagent kits. CON: distilled water, EtOH: 40% EtOH, 2.5 mL/kg, ECL: EtOH + Cii low concentration, 2 mg/kg, ECH: EtOH + Cii high concentration, 10 mg/kg, and ES: EtOH + silymarin, 100 mg/kg. Data were expressed as mean ± S.E.M. (*n* = 7). ^#^*P* < 0.001, CON vs. EtOH; ^*∗∗∗*^*P* < 0.001, EtOH vs. ECL; ^*∗∗*^*P* < 0.005, EtOH vs. ECH and ES. (a) Serum GOP level. (b) Serum GPT level. (c) Serum TG level. (d) Liver TG level.

To assess the effects of Cii on liver function, the levels of serum biomarkers GOT and GPT were measured in the long-term administration experiment. The levels of both were significantly higher in the EtOH group compared with the normal group. Treatment with Cii at 2 and 10 mg/kg significantly suppressed alcohol-induced increases in serum GOT and GPT levels compared with the EtOH group. The levels were 27.60 ± 2.93, 155.40 ± 7.39, 111.00 ± 9.04, 49.21 ± 0.56, and 46.63 ± 7.78 IU/L for serum GOT in the CON, EtOH, ECL, ECH, and ES groups, respectively (Figure 7(a)), and 22.68 ± 1.46, 166.10 ± 19.52, 94.53 ± 9.55, 36.30 ± 3.30, and 29.32 ± 4.74 IU/L for serum GPT (Figure 7(b)). The results showed that Cii can inhibit the increases of serum GOT and GPT levels in rats with alcohol-induced liver injury in a dose-dependent manner. To quantitatively confirm the effect of Cii on alcohol-induced lipid accumulation, the TG contents in serum and liver tissues were measured in the long-term administration experiment. Treatment with Cii at 2 and 10 mg/kg did not significantly reduce alcohol-induced increase in the TG content in serum compared with the EtOH group (Figure 7(c)). The content of TG in liver tissues was significantly higher in the EtOH group compared with the normal group. Treatment with Cii at 2 and 10 mg/kg significantly reduced alcohol-induced increase in the liver tissue TG content compared with the EtOH group. The liver TG levels were 91.39 ± 9.74, 535.00 ± 35.83, 301.40 ± 21.65, 120.20 ± 38.66, and 84.98 ± 13.20 ng/*μ*L in the CON, EtOH, ECL, ECH, and ES groups, respectively (Figure 7(d)). The results showed that Cii can decrease the content of TG and inhibit hepatic lipid accumulation in rats with alcohol-induced liver injury.

### 3.7. Effects of Cii on Alcoholysis Enzymes in Rats with Alcohol-Induced Injury

**Figure 8:**
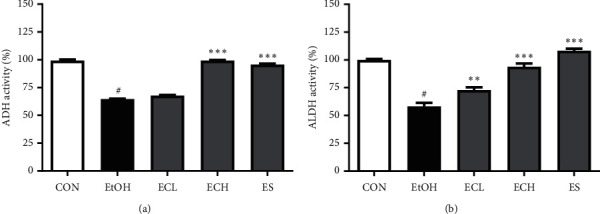
Effects of Cii on ADH and ALDH activities in liver tissues of alcohol-induced rats. The activity of ADH and ALDH in liver tissues was determined by commercial reagent kits. CON: distilled water, EtOH: 40% EtOH, 2.5 mL/kg, ECL: EtOH + Cii low concentration, 2 mg/kg, ECH: EtOH + Cii high concentration, 10 mg/kg, and ES: EtOH + silymarin, 100 mg/kg. Data were expressed as mean ± S.E.M. (*n* = 7). ^#^*P* < 0.001, CON vs. EtOH; ^*∗∗*^*P* < 0.005, EtOH vs. ECL; ^*∗∗∗*^*P* < 0.001, EtOH vs. ECH and ES. (a) ADH activity. (b) ALDH activity.

The activity of ADH and ALDH, the most important enzymes in body alcohol metabolism, was investigated to examine the alcohol decomposition activity of Cii. Activity of ADH, which is an alcohol-degrading enzyme, was significantly impaired in the alcohol group compared with the normal group, and a concentration-dependent increase in the Cii group was observed. The ADH activity was 64.66 ± 0.36, 67.81 ± 0.44, 99.30 ± 0.45, and 95.74 ± 0.81% in the EtOH, ECL, ECH, and ES groups, respectively (Figure 8(a)). Similarly, the ALDH activity was significantly impaired in the alcohol group compared with the normal group, and a concentration-dependent increase in the Cii group was evident. The ALDH activity was 58.13 ± 3.35, 72.87 ± 2.41, 93.99 ± 2.86, and 108.30 ± 1.75% in the EtOH, ECL, ECH, and ES groups, respectively (Figure 8(b)).

### 3.8. Effects of Cii on the Expression of CYP2E1 in Liver Tissues of Rats with Alcohol-Induced Liver Injury

**Figure 9:**
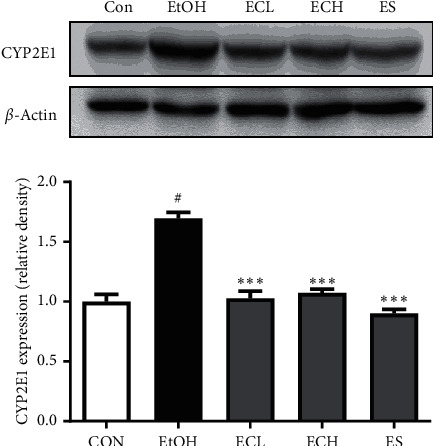
Effects of Cii on the expression of CYP2E1 in liver tissues of alcohol-induced rats. Expression of CYP2E1 in liver tissues was examined by western blot. The relative expression of CYP2E1 protein was normalized to that of *β*-actin. CON: distilled water, EtOH: 40% EtOH, 2.5 mL/kg, ECL: EtOH + Cii low concentration, 2 mg/kg, ECH: EtOH + Cii high concentration, 10 mg/kg, and ES: EtOH + silymarin, 100 mg/kg. The results were an average of 4 similar experiments, expressed as mean ± S.E.M. The inserts displayed representative blots of four similar independent experiments. ^#^*P* < 0.001, CON vs. EtOH; ^*∗∗∗*^*P* < 0.001, EtOH vs. ECL, ECH, or ES.

As CYP2E1 is a major contributor to the production of reactive oxygen species (ROS), we examined the expression of CYP2E1 in liver tissues using western blot analyses. The results indicated that treatment with Cii significantly inhibited ethanol-induced upregulation of CYP2E1 expression and that Cii can produce antioxidative stress by inhibiting the CYP2E1 expression in liver tissues of rats with ethanol-induced liver injury. The relative CYP2E1 density was 1.69 ± 0.05, 1.03 ± 0.06, 1.07 ± 0.03, and 0.90 ± 0.04 in the EtOH, ECL, ECH, and ES group, respectively (Figure 9).

### 3.9. Effects of Cii on Liver Histopathology in Rats with Alcohol-Induced Injury

**Figure 10:**
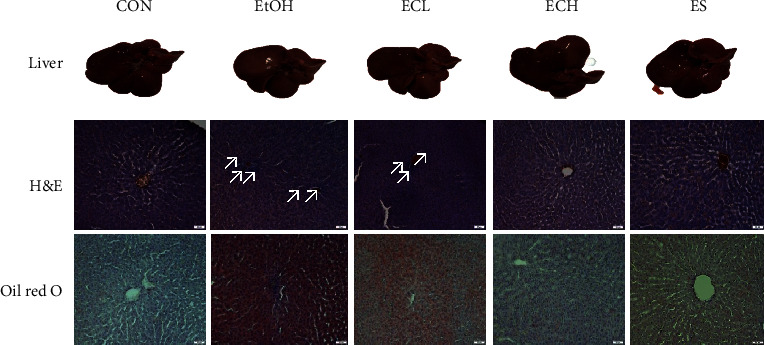
Effects of Cii on liver histopathology in alcohol-induced rats. The cryotissues were sectioned and stained. Some sections were cut into slices (20 *μ*m thick) and stained with hematoxylin and eosin (H&E). The others were then cut into slices (17 *μ*m thick) and stained with oil red O. Histopathological changes of liver tissues were visualized with an Olympus DP80 microscope. CON: distilled water, EtOH: 40% EtOH, 2.5 mL/kg, ECL: EtOH + Cii low concentration, 2 mg/kg, ECH: EtOH + Cii high concentration, 10 mg/kg, and ES: EtOH + silymarin, 100 mg/kg. ×200 magnification.

To directly evaluate the protective effects of Cii on alcohol-induced liver injury, liver histopathology was analyzed. Representative sections of rat livers stained with H&E and oil red O are shown in Figure 10. The liver tissues were intact, the hepatic lobules were clear, and the hepatocytes were arranged regularly in the control group. In the EtOH group, the basic architecture of liver cells was lost, while inflammatory cell infiltration and liver cell swelling were evident. In contrast, treatment with Cii significantly ameliorated the degree of histopathological alterations, particularly in the silymarin and 10 mg/kg Cii groups. Only slight fatty degeneration was seen in Cii groups. Staining with oil red O revealed fatty deposition in rat liver tissues. Rats in the control group had no red lipid droplets in the liver tissue. The EtOH group exhibited diffuse and granular accumulation of hepatocyte lipid droplets that merged in confluence. Markedly fewer scattered and sparse lipid droplets were evident in the Cii hepatocytes compared with the EtOH group. Compared with the EtOH group, the number of lipid droplets in the Cii and silymarin group hepatocytes was significantly reduced, and the droplets were scattered and sparse. In addition, the droplets were relatively small and unevenly distributed (Figure 10).

## 4. Discussion

This study was conducted to investigate the protective effect of Cii in alcohol-induced liver injury. Alcohol is absorbed in the stomach and small intestine immediately after consumption and metabolized primarily in the liver, some of which is excreted through urine and sweat [15, 16]. Alcohol is a cause of headaches and can affect neurotransmitters and hormones [17]. Acetaldehyde is a hangover-inducing substance and is toxic, leading to liver disease associated with mitochondrial dysfunction and decreased activity of ALDH in combination with the components in the body due to its wide chemical reactivity [18].

Animal models of ALD are used widely in the study of alcohol-induced liver damage. This study established an experimental animal model through administration of alcohol and explored the protective effect and basic mechanism of Cii against associated liver damage. The data confirmed that Cii provides a potentially protective effect against liver damage induced by alcohol by hindering antioxidant stress and lipid accumulation through the ADH and CYP2E1 pathways [19].

The apoptosis rate of cells tended to decrease at extract concentrations of 50–400 *μ*g/mL, suggesting that Cii can inhibit cell death caused by alcohol (Figure 2(b)). This analysis showed a higher degree of DPPH radical erasing with Cii increase, suggesting a concentration-dependent antioxidant ability of Cii (Figure 4).

To measure the hangover improvement and liver protection effects of Cii, several markers were identified in blood and liver tissues. First, in short-term administration, the blood-alcohol level tended to decrease faster in Cii groups than in alcohol groups (Figures 5(a) and 5(b)). These results suggest that Cii promotes fast decomposition of alcohol. We established a chronic-binge alcohol model in rats and explored the protective effects and underlying mechanisms responsible for the effects. In long-term administration, blood-alcohol concentration was significantly higher in the alcohol group compared with the normal group and significantly lower in the ECH group compared with the alcohol group, suggesting that Cii promotes alcohol decomposition (Figure 5(c)). The weight gain in each experimental group was lower in the EtOH and ECL groups than in the normal group (Figure 6(a)). Lieber and Oh et al. reported that alcohol intake hinders animal growth [20, 21]. Gruchow et al. and Pikaar et al. reported that this results in reduced food intake due to the calorific content of alcohol consumed and increased metabolic rates, eventually leading to weight loss due to decreased ATP production of alcohol-oxidizing components in microsomes [22, 23]. The weight of the liver was divided by the total weight to minimize the differences in weight among livers. One of the early symptoms of chronic EtOH intake is liver cirrhosis, resulting in accumulation of fat, moisture, and protein in the liver cell cytosol, which can produce liver hyperplasia [24]. Liver weight tended to increase in the EtOH group compared with the normal group, explaining the decrease in the ECH group. This suggests that Cii inhibits hyperplasia in alcohol-induced liver injury (Figure 6(b)). GOT, which is also known as aspartate aminotransferase, is an enzyme that promotes transamination. GPT, also known as alanine aminotransferase, is an enzyme that produces glutamic and pyruvic acids in response to alanine and *α*-ketoglutaric acid and can also be produced in liver injury or long-term exercise. Damage to liver cells causes changes in transport function and membrane permeability, releasing enzymes such as GOT and GPT from liver cells into blood, with the activity in serum proportional to the degree of liver damage. Therefore, serum GOT and GPT levels are important and sensitive biochemical hallmarks of liver function and provide an early indication of ALD. Abnormal increases in their levels can cause injury and necrosis of liver cells [25].

In the long-term administration experiment, treatment with Cii markedly decreased the increased levels of GOT and GPT induced by alcohol, indicating that Cii can reduce alcohol-induced liver injury by stabilizing hepatocyte membranes (Figures 7(a) and 7(b)). In the short-term administration experiment, treatment with Cii significantly suppressed alcohol-induced serum GOT and GPT levels (data not shown). Chronic or excessive alcohol intake increases the production of NADH/NAD+, resulting in metabolic disturbances in carbohydrates, fats, and proteins [26, 27]. In particular, oxidation of fatty acids in the liver is inhibited, while synthesis of fatty acids is increased, producing a fatty liver. The liver synthesizes neutral fats using fatty acids of blood and releases neutral fat to blood if necessary [28]. Damage to liver tissues caused by long-term alcohol intake inhibits the outflow of liver fat, reducing the concentration of neutral fat and other fats in blood and inducing accumulation in the liver tissue. A fat liver is an early symptom of liver toxicity caused by excessive alcohol consumption and causes oxygen and nutrient imbalance in liver cells. In serum, the TG content increased significantly in the alcohol group compared with the normal group but did not change significantly in the drug group (Figure 7(c)). However, the TG content in liver tissues was significantly higher in the alcohol group compared with the normal group, indicating a significant decrease in the Cii group compared with the alcohol group (Figure 7(d)). Treatment with Cii decreased the increased TG content induced by alcohol in the liver, indicating that the extract can improve hepatocyte steatosis and prevent the development of the fatty liver. This result is consistent with histopathological observations. Alcohol metabolism in the liver is carried out mainly by ADH and ALDH, which are NAD-linked enzymes. ADH, which uses NAD+ as a coenzyme, converts alcohol into acetaldehyde that is excreted as acetic acid and CO_2_ by ALDH. Acetate is converted into acetyl CoA and used to generate energy through the TCA cycle or to synthesize cholesterol and fatty acids. The activity of ADH and ALDH was investigated to examine the alcohol decomposition activity of Cii. Activity of ADH was significantly impaired in the alcohol group compared with the normal group, and a concentration-dependent increase in the Cii group was observed (Figure 8(a)). Similarly, ALDH activity was significantly higher in the alcohol group compared with the normal group, and a concentration-dependent increase in the Cii group was observed (Figure 8(b)). This indicates that Cii increases ADH and ALDH activities and that the extract increases the cellular ability to decompose alcohol. Cii increases ADH and ALDH activities to inhibit liver damage by alcohol. CYP2E1 is one of the main members of the cytochrome P450 family, a major enzyme system involved in metabolism in organisms. CYP2E1 is the most relevant of the family to ALD because of its high inducibility and high catalytic activity [29]. CYP2E1 is present primarily in liver microsomes and plays a critical role in ROS production and liver injury. Acute and chronic alcohol intake increases the activity of CYP2E1, which catalyzes the conversion of alcohol to acetaldehyde and results in excessive ROS production [30]. Excessive ROS can cause oxidative stress in the liver, while suppressing the antioxidant stress defense pathway [31]. Treatment with Cii significantly inhibited alcohol-induced expression of CYP2E1 in the liver, which indicates that the protective mechanism of Cii on alcohol-induced liver injury can be at least partially attributed to the potential antioxidative stress produced by suppressing the CYP2E1 expression (Figure 9).

Staining of the liver was lighter in the alcohol group than in the others [32]. H&E staining displayed a uniform arrangement of uniformly shaped cells in the control group. In comparison, the alcohol group showed swelling and modification of liver cells around the blood vessels. In the chicory treatment group, these hepatic lesions were reduced in a concentration-dependent manner. Oil red O staining suggests that Cii inhibits the alcohol-induced accumulation of lipids in liver cells.

This study revealed reduced alcohol-induced liver injury in Cii treatment groups and discussed the role of Cii in ALD. The results suggest that Cii can be useful in reducing liver damage due to alcohol intake by reducing the TG level and increasing the activity of the antioxidant and alcohol-degrading enzymes. The extract can delay or mitigate liver damage and liver disease. Further clinical trial studies are required to determine the precise link between Cii and alcohol-induced liver injury.

## Data Availability

The data used to support the findings of this study are available from the corresponding author upon request.

## References

[1] Lee S.-E., Lee J.-H., Kim G.-S. (2012). Effect of root extract of Lythrum salicaria L. on liver function of rat acutely administrated with alcohol. *Korean Journal of Medicinal Crop Science*.

[2] Stickel F., Datz C., Hampe J., Bataller R. (2017). Pathophysiology and management of alcoholic liver disease: update 2016. *Gut and Liver*.

[3] Jameson J. L. (2018). *Harrison’s Principles of Internal Medicine*.

[4] Xu L., Yu Y., Sang R., Li J., Ge B., Zhang X. (2018). Protective effects of taraxasterol against ethanol-induced liver injury by regulating CYP2E1/Nrf2/HO-1 and NF-*κ*B signaling pathways in mice. *Oxidative Medicine and Cellular Longevity*.

[5] Du J., He D., Sun L.-N. (2020). Semen Hoveniae extract protects against acute alcohol-induced liver injury in mice. *Pharmaceutical Biology*.

[6] Ronis M., Huang J., Crouch J. (1993). Cytochrome P450 CYP 2E1 induction during chronic alcohol exposure occurs by a two-step mechanism associated with blood alcohol concentrations in rats. *Journal of Pharmacology And Experimental Therapeutics*.

[7] Li S., Wang N., Hong M., Tan H.-Y., Pan G., Feng Y. (2019). Hepatoprotective effects of a functional formula of three Chinese medicinal herbs: experimental evidence and network pharmacology-based identification of mechanism of action and potential bioactive components. *Molecules*.

[8] Lieber C. S. (1994). Alcohol and the liver: 1984 update. *Hepatology*.

[9] Nagappan A., Kim J.-H., Jung D. Y., Jung M. H. (2019). Cryptotanshinone from the salvia miltiorrhiza bunge attenuates ethanol-induced liver injury by activation of AMPK/SIRT1 and Nrf2 signaling pathways. *International Journal Of Molecular Sciences*.

[10] Rouach H., Clément M., Orfanelli M.-T., Janvier B., Nordmann J., Nordmann R. (1983). Hepatic lipid peroxidation and mitochondrial susceptibility to peroxidative attacks during ethanol inhalation and withdrawal. *Biochimica et Biophysica Acta (BBA)-Lipids and Lipid Metabolism*.

[11] Moncada C., Torres V., Varghese G., Albano E., Israel Y. (1994). Ethanol-derived immunoreactive species formed by free radical mechanisms. *Molecular Pharmacology*.

[12] Nagappan A., Jung D. Y., Kim J.-H., Lee H., Jung M. H. (2018). Gomisin N alleviates ethanol-induced liver injury through ameliorating lipid metabolism and oxidative stress. *International Journal of Molecular Sciences*.

[13] Shi C., Wang Y., Gao J. (2017). Inhibition of aldose reductase ameliorates alcoholic liver disease by activating AMPK and modulating oxidative stress and inflammatory cytokines. *Molecular Medicine Reports*.

[14] Shin S., Park S.-S., Lee H.-M., Hur J.-M. (2021). Effects of fermented chicory fiber on the improvement of intestinal function and constipation. *Journal of the Korean Society of Food Science and Nutrition*.

[15] Ohashi K., Pimienta M., Seki E. (2018). Alcoholic liver disease: a current molecular and clinical perspective. *Liver Research*.

[16] Kim N. H., Sung S. H., Heo J. D., Jeong E. J. (2015). The extract of Limonium tetragonum protected liver against acute alcohol toxicity by enhancing ethanol metabolism and antioxidant enzyme activities. *Natural Product Sciences*.

[17] Chen C., Wen D. C., Gao S. D., Hu X. Y., Yi C. (2016). The protective effects of Buzui on acute alcoholism in mice. *Evidence-Based Complementary and Alternative Medicine: ECAM*.

[18] Lee J.-S., Kim N.-Y., Lee K.-H. (2020). Effects of flower of Pueraria lobata on lipid peroxidation and activities of alcohol metabolic enzymes in alcohol-treated rats. *Joural Korean Society of Food Science and Nutrition*.

[19] Louvet A., Mathurin P. (2015). Alcoholic liver disease: mechanisms of injury and targeted treatment. *Nature Reviews. Gastroenterology and Hepatology*.

[20] Lieber C. S. (1994). Alcohol and the liver: 1994 update. *Gastroenterology*.

[21] Cha Y.-S., Choi D.-S., Oh S.-H. (1999). Effects of Angelica gigas Nakai diet on lipid metabolism, alcohol metabolism and liver function of rats administered with chronic ethanol. *Applied Biological Chemistry*.

[22] Gruchow H. W., Sobocinski K. A., Barboriak J. J., Scheller J. G. (1985). Alcohol consumption, nutrient intake and relative body weight among US adults. *The American Journal of Clinical Nutrition*.

[23] Pikaar N. A., Wedel M., Van Der Beek E. J. (1998). Effects of moderate alcohol consumption on platelet aggregation, fibrinolysis, and blood lipids. *Metabolism*.

[24] Lieber C. S. (1985). Alcohol and the liver: metabolism of ethanol, metabolic effects and pathogenesis of injury. *Acta Medica Scandinavica*.

[25] Cao Y.-W., Jiang Y., Zhang D.-Y. (2015). Protective effects of penthorum Chinense pursh against chronic ethanol-induced liver injury in mice. *Journal of Ethnopharmacology*.

[26] Mezey E. (1980). Alcoholic liver disease: roles of alcohol and malnutrition. *The American Journal of Clinical Nutrition*.

[27] Lieber C. S. (2000). Alcohol and the liver: metabolism of alcohol and its role in hepatic and extrahepatic diseases. *The Mount Sinai Journal of Medicine*.

[28] Zeman F. *Liver Disease and Alcoholism in Clinical Nutrition and Dietetics*.

[29] Lieber C. S. (1997). Cytochrome P-4502E1: its physiological and pathological role. *Physiological Reviews*.

[30] Jimenez-Lopez J. M., Cederbaum A. I. (2003). CYP2E1-dependent oxidative stress and toxicity: role in ethanol-induced liver injury. *Expert Opinion on Drug Metabolism and Toxicology*.

[31] Leung T.-M., Nieto N. (2013). CYP2E1 and oxidant stress in alcoholic and non-alcoholic fatty liver disease. *Journal of Hepatology*.

[32] Kong X., Yang Y., Ren L. (2017). Activation of autophagy attenuates EtOH-LPS-induced hepatic steatosis and injury through MD2 associated TLR4 signaling. *Scientific Reports*.

